# Screening of Bifidobacteria and Lactobacilli Able to Antagonize the Cytotoxic Effect of *Clostridium difficile* upon Intestinal Epithelial HT29 Monolayer

**DOI:** 10.3389/fmicb.2016.00577

**Published:** 2016-04-22

**Authors:** Lorena Valdés-Varela, Marta Alonso-Guervos, Olivia García-Suárez, Miguel Gueimonde, Patricia Ruas-Madiedo

**Affiliations:** ^1^Department of Microbiology and Biochemistry of Dairy Products, Instituto de Productos Lácteos de Asturias–Consejo Superior de Investigaciones CientíficasVillaviciosa, Spain; ^2^Optical Microscopy and Image Processing Unit, University Institute of Oncology of Asturias, Scientific-Technical Services, University of OviedoOviedo, Spain; ^3^Department of Morphology and Cellular Biology, University of OviedoOviedo, Spain

**Keywords:** probiotics, *Clostridium difficile*, toxins, RTCA, xCelligence, *Bifidobacterium*, *Lactobacillus*, microscopy

## Abstract

*Clostridium difficile* is an opportunistic pathogen inhabiting the human gut, often being the aetiological agent of infections after a microbiota dysbiosis following, for example, an antibiotic treatment. *C. difficile* infections (CDI) constitute a growing health problem with increasing rates of morbidity and mortality at groups of risk, such as elderly and hospitalized patients, but also in populations traditionally considered low-risk. This could be related to the occurrence of virulent strains which, among other factors, have high-level of resistance to fluoroquinolones, more efficient sporulation and markedly high toxin production. Several novel intervention strategies against CDI are currently under study, such as the use of probiotics to counteract the growth and/or toxigenic activity of *C. difficile*. In this work, we have analyzed the capability of twenty *Bifidobacterium* and *Lactobacillus* strains, from human intestinal origin, to counteract the toxic effect of *C. difficile* LMG21717 upon the human intestinal epithelial cell line HT29. For this purpose, we incubated the bacteria together with toxigenic supernatants obtained from *C. difficile*. After this co-incubation new supernatants were collected in order to quantify the remnant A and B toxins, as well as to determine their residual toxic effect upon HT29 monolayers. To this end, the real time cell analyser (RTCA) model, recently developed in our group to monitor *C. difficile* toxic effect, was used. Results obtained showed that strains of *Bifidobacterium longum* and *B. breve* were able to reduce the toxic effect of the pathogen upon HT29, the RTCA normalized cell-index values being inversely correlated with the amount of remnant toxin in the supernatant. The strain *B. longum* IPLA20022 showed the highest ability to counteract the cytotoxic effect of *C. difficile* acting directly against the toxin, also having the highest capability for removing the toxins from the clostridial toxigenic supernatant. Image analysis showed that this strain prevents HT29 cell rounding; this was achieved by preserving the *F*-actin microstructure and tight-junctions between adjacent cells, thus keeping the typical epithelium-like morphology. Besides, preliminary evidence showed that the viability of *B. longum* IPLA20022 is needed to exert the protective effect and that secreted factors seems to have anti-toxin activity.

## Introduction

*Clostridium difficile* is a Gram-positive, spore-forming, motile and strict anaerobe rod that can be found in the gastrointestinal tract of humans and animals (Janezic et al., 2014). The current classification of the “Bergey’s Manual of Systematic Bacteriology” includes *C. difficile* in the Phylum *Firmicutes*, Class *Clostridia*, Order *Clostridiales* and Family *Peptostreptococcaceae* (Ludwig et al., 2009). A recent taxonomic study, based on 16S rRNA and ribosomal protein sequences, ascertains that *C. difficile* belongs to this family and proposes that it should be renamed as *Peptoclostridium difficile* (Yutin and Galperin, 2013); this new name appears in the taxonomic classification and nomenclature catalog of NCBI^1^, but still *C. difficile* remains as the name recognized by the clinical and scientific community.

*C. difficile* infection (CDI) is the main cause of diarrhea associated with antibiotic use or related to health-care environments (Leffler and Lamont, 2015) and increasing incidence is reported among populations previously considered as low risk, such as pregnant women and children (Carter et al., 2012). The ubiquity of this bacterium, in combination with its capability to form spores, makes hospital environments a good source for *C. difficile* acquisition, although zoonotic (Bauer and Kuijper, 2015) and food transmissions (Troiano et al., 2015) have been proposed as well. The incidence and severity of CDI has been growing since the beginning of this century due to the global occurrence of hypervirulent strains such as BI/NAP1/027 (group BI by restriction endonuclease analysis, North American pulse-field type NAP1 by pulse-field gel electrophoresis, and ribotype 027; Rupnik et al., 2009; Yakob et al., 2015). The antibiotics metronidazole and vancomycin are the current treatments for CDI, but this does not prevent the high rates of recurrence. Thus, new emerging therapeutic options, such as fecal microbiota transplantation (FMT), new antibiotics, bacteriocins, bacteriophages, and probiotics are under evaluation for the control of CDI (Martin et al., 2013; Dunne et al., 2014; Mathur et al., 2015). Indeed probiotics, which are “live microorganisms that, when administered in adequate amounts, confer a health benefit on the host” (FAO-WHO, 2001; Hill et al., 2014), have been proposed as biotherapeutic agents to help microbiota restoration after a dysbiosis caused by antibiotics or infections (Reid et al., 2011).

The information encoded on the genomes of this species, excellently reviewed by Knight et al. (2015), reveals high plasticity and very low levels of conservation among strains. This genetic diversity is reflected in its physiological adaptation to different ecosystems and in the occurrence of different phenotypes. In addition, the presence of a wide variety of transposons and phages explain the lineage evolution of clinically relevant loci, such as the antimicrobial resistance genes and the PaLoc (pathogenicity locus), among others (Knight et al., 2015). The PaLoc harbors, together with three additional genes, *tcdA* and *tcdB* coding for toxin A and toxin B, respectively, which are the major *C. difficile* virulence factors (Monot et al., 2015). The modulating environmental signals regulating the expression of PaLoc is not totally understood and a recent report shows that toxin synthesis is regulated through quorum-sensing signaling (Darkoh et al., 2015). TcdA and TcdB are large toxins whose main mechanism of action is known, although host receptors and toxin-mediated responses still remain to be fully deciphered. They act as intracellular glycosyltransferases modifying the Ras superfamily of small GTPases thus inducing intracellular changes, including *F*-actin condensation, transcriptional activation and cell apoptosis of intestinal epithelial cells. This promotes the disruption of the tight junctions and barrier integrity, leading to an increase in the gut permeability and neutrophil infiltration. Downstream effects also include modifications in the chemokine and cytokine production patterns toward an inflammatory response and fluid accumulation, ending with the clinical manifestations of leukocytosis and diarrhea (Voth and Ballard, 2005; D’Auria et al., 2013; Carter et al., 2015; Leslie et al., 2015). Therefore, anti-toxin therapies to counteract the negative effects of these potent *C. difficile* virulence factors could be valuable tools to reduce the course of CDI (Tam et al., 2015).

In a previous study we developed a biological model, using the (human) intestinal epithelial cell line HT29, to follow in real time the effect of supernatants collected from *C. difficile* cultures of a TcdA+, TcdB+ (toxinotype 0) strain. This method is based on the continuous monitoring of the impedance signal, transmitted through gold microelectrodes placed in the bottom of microtiter plates, of HT29 monolayers (Valdés et al., 2015). Our aim in the present work is to search for lactobacilli and bifidobacteria probiotic candidates with anti-toxin capability able to protect HT29 cells from the cytotoxicity caused by toxigenic *C. difficile* supernatants.

## Materials and Methods

### Bacterial Strains and Culture Conditions

The *Bifidobacterium* and *Lactobacillus* species used in this study are listed in **Table 1**. Most strains belonging to IPLA culture collection were isolated from infant feces and breast milk (Solís et al., 2010), whereas IPLA20031 and IPLA20032 were obtained after adaptation to increasing concentrations of bile salts from a parental strain isolated from a dairy product (Ruas-Madiedo et al., 2010). Strains were grown in MRSC [MRS (Biokar Diagnostics, Beauvois, France) supplemented with 0.25% L-cysteine (Sigma-Chemical Co., St. Louis, MO, USA)] at 37°C in the anaerobic chamber MG500 (Don Whitley Scientific, Yorkshire, UK) under 80% N_2_, 10% CO_2_ and 10% H_2_ atmosphere. As standard procedure bacterial stocks, kept at -80°C in MRSC + 20% glycerol, were spread onto the surface of agar-MRSC and incubated for 3 days. A single colony was picked to inoculate MRSC broth which, after 24 h incubation, was used to inoculate (2%) 10 ml fresh MRSC broth. This culture was incubated overnight (18 h) to prepare the bacterial suspensions that will be described next.

**Table 1:** Strains included in this study and normalized cell index (CI) obtained at 4 and 22 h after addition of neutralized cell-free supernatants (NCFS) collected from incubations of each bifidobacteria or lactobacilli strain with 2.5% of toxigenic *Clostridium difficile* LGM21717 supernatant (Tox-S).

		Mean ± SD			
		
		Normalized-CI		Remnant toxin (ng/ml)	
		
NCFS	Strain	After 4 h	After 22 h	TcdA	TcdB
*C. difficile* Tox-S (2.5%)	LMG21717^∗^	-0.64 ± 0.13	-0.93 ± 0.11	4.41 ± 0.01	0.48 ± 0.0
*B. bifidum*	LMG13195^∗^	-0.62 ± 0.13	-0.97 ± 0.10	4.05 ± 0.33	0.15 ± 0.01
	IPLA20024	-0.43 ± 0.12	-0.92 ± 0.10	2.87 ± 0.02	0.11 ± 0.01
	IPLA20025	-0.64 ± 0.08	-1.03 ± 0.07	3.47 ± 0.09	0.14 ± 0.02
	IPLA20017	-0.71 ± 0.11	-1.04 ± 0.07	3.50 ± 0.12	0.42 ± 0.01
*B. animalis*	DSM15954^†^ (Bb12)	-0.58 ± 0.09	-1.00 ± 0.13	3.71 ± 0.33	0.41 ± 0.04
	IPLA20031 (A1dOx)	-0.53 ± 0.05	-1.04 ± 0.07	4.60 ± 0.48	0.58 ± 0.26
	IPLA20032 (A1dOxR)	-0.64 ± 0.11	-1.00 ± 0.12	4.32 ± 0.3	0.42 ± 0.03
	IPLA20020	-0.62 ± 0.07	-1.05 ± 0.08	4.08 ± 0.42	0.42 ± 0.04
*B. longum*	IPLA20021	-0.16 ± 0.11	-0.60 ± 0.20	1.50 ± 0.14	0.29 ± 0.14
	IPLA20022	-0.06 ± 0.05	-0.06 ± 0.12	0.54 ± 0.18	0.26 ± 0.04
	IPLA20001	-0.09 ± 0.06	-0.33 ± 0.19	1.71 ± 0.05	0.26 ± 0.04
	IPLA20002	-0.07 ± 0.08	-0.57 ± 0.09	1.25 ± 0.16	0.22 ± 0.05
*B. breve*	IPLA20004	-0.00 ± 0.04	-0.24 ± 0.12	0.75 ± 0.09	0.21 ± 0.08
	IPLA20005	-0.03 ± 0.03	-0.25 ± 0.09	0.56 ± 0.29	0.15 ± 0.01
	IPLA20006	-0.12 ± 0.01	-0.66 ± 0.08	1.05 ± 0.37	0.12 ± 0.01
*B. pseudocatenulatum*	IPLA20026	-0.45 ± 0.06	-0.91 ± 0.13	3.31 ± 0.02	0.40 ± 0.12
*L. crispatus*	IPLA20120	-0.44 ± 0.06	-0.91 ± 0.00	4.09 ± 1.64	0.22 ± 0.09
*L. gasseri*	IPLA20121	-0.42 ± 0.04	-0.91 ± 0.03	3.68 ± 1.28	0.43 ± 0.16
*L. paracasei*	IPLA20124	-0.39 ± 0.03	-0.83 ± 0.03	4.57 ± 0.26	0.46 ± 0.03
*L. rhamnosus*	LMG18243^∗^ (GG)	-0.41 ± 0.03	-0.83 ± 0.03	3.35 ± 0.44	0.42 ± 0.04


*^∗^LMG: Belgian Coordinated Collections of Microorganisms” (BCCM, Gent, Belgium).*

*^†^DSM: German Collection of Microorganisms and Cell cultures (DSMZ, Braunschweig, Germany).*

*The remnant toxins in these NCFS were quantified by ELISA tests. Data were obtained from two biological replicates each measured by duplicate.*

The strain *C. difficile* LMG21717 (∼ATCC9689, Ribotype 001, genes *tcdA*+, *tcdB*+, *cdtB*-) producing both TcdA and TcdB toxins (Toxinotype 0) was purchased from the “Belgian Coordinated Collections of Microorganisms” (BCCM, Gent, Belgium). The strain was routinely grown in Reinforced Clostridium Medium (RCM, Oxoid, Thermo Fisher Scientific Inc., Waltham, MA, USA) in Hungate tubes under anaerobic conditions at 37°C. Frozen stocks (-80°C in RCM + 20% glycerol) were directly activated in RCM broth incubated for 24 h and this culture was used to inoculate (2%) fresh medium that was cultivated for 13 h. This culture was used as inoculum to obtain the toxigenic supernatant.

#### Preparation of Toxigenic *C. difficile* Supernatant

Conditions to obtain toxigenic supernatant from *C. difficile* LMG21717 have previously been determined and published (Valdés et al., 2015). In short, 300 μl of RCM grown culture were used to inject into Hungates tubes containing 15 ml of Gifu Anaerobic Medium (GAM, Nissui Pharmaceutical Co., Ltd., Tokyo, Japan). GAM cultures were incubated for 48 h and centrifuged (16,000 × *g*, 10 min) to obtain the *C. difficile-*free toxigenic supernatant (Tox-S), which was kept in several aliquots at -80°C.

Two independent ELISA tests (tgcBIOMICS GmbH, Bingen, Germany) were used to quantify the concentration of TcdA or TcdB in the toxigenic supernatant, as well as the remnant toxins in the neutralized bacterial-supernatants obtained after incubation of Tox-S with bifidobacteria and lactobacilli.

#### Incubation of Bifidobacteria or Lactobacilli with Toxigenic *C. difficile* Supernatant

The experimental design carried out in this study is schematized in **Supplementary Figure S1A**. Bifidobacteria and lactobacilli cultures grown for 18 h in MRSC were washed twice with PBS and resuspended at 10^9^ cfu/ml in the HT29-cultivation medium (MM, described below) supplemented with 5% of Tox-S from *C. difficile* or without supplementation (controls). After incubation for 1 h under anaerobic conditions and mild stirring (∼300 rpm), the bacterial suspensions were centrifuged (16,000 × *g*, 10 min) to obtain bifidobacteria- or lactobacilli-free bacterial supernatants. Then, the pH was increased to 7.55 ± 0.05 with 1 and 0.1 N NaOH and the volume obtained was adjusted to twice the initial one with MM; this means that the maximum amount of remnant toxin that could be present was 2.5%. These neutralized cell-free supernatants (NCFS) were directly used to test their cytotoxicity upon HT29 monolayers as well as to quantify the remnant TcdA and TcdB toxins. This screening was performed with two biological replicates, each analyzed in duplicate, of each bacterial strain using HT29 monolayers of two consecutive passages (p147 and p148).

#### Incubation of Dead and Live *B. longum* IPLA20022 with Toxigenic *C. difficile* Supernatant

The strain *B. longum* IPLA20022 was selected in order to determine whether the capability to diminish the cytotoxic effect of *C. difficile* supernatant was dependent on bacterial viability. For that purpose UV-treated IPLA20022 suspensions were prepared from MRSC-grown cultures that were washed and resuspended in PBS at 10^9^ cfu/ml. Then, the PBS suspension was poured into several petri dishes allowing a high surface spread and they were submitted to ultra violet radiation in a UV-chamber (15W, Selecta, Barcelona, Spain). Three UV cycles of 30 min were applied, homogenizing the PBS suspension in each interval, and the absence of viability was checked by plating serial dilutions of UV-treated IPLA20022 suspension in agar-MRSC (López et al., 2012). Incubation of this UV-treated suspension (dead IPLA20022) with toxigenic *C. difficile* supernatant was performed as previously described. A non UV-killed suspension (live IPLA20022) of the same culture was used as control. After incubation for 1 h, both suspensions were processed to obtain the respective NCFS (**Supplementary Figure S1A**). This experiment was carried out with three independent cultures (biological replicates) of strain IPLA20022 upon HT29 monolayers within the same passage (p149), each measured in duplicate.

#### Incubation of Supernatants from *B. longum* IPLA20022 with Toxigenic *C. difficile* Supernatant

To test the activity of putative secreted factors by *B. longum* IPLA20022 against *C. difficile* toxins, cell-free bifidobacterial supernatants obtained from three independent-culture replicates (each analyzed in duplicate) were incubated with 50% toxigenic (Tox-S) supernatant for 1 h under anaerobic conditions. Afterward, supernatants were neutralized (pH ≥ 7.5) and its cytotoxic activity tested upon HT29 monolayers (passage p149) at 2.5% in MM (**Supplementary Figure S1B**).

### Intestinal Epithelial Cell Line HT29 and Culture Conditions

The intestinal cell line HT29 (ECACC 91072201), from human colon adenocarcinoma, was purchased from the “European Collection of Cell Cultures” (Salisbury, UK) and stored at IPLA under liquid N_2_. McCoy’s Medium (MM) supplemented with 10% foetal bovine serum (FBS), 3 mM L-glutamine and a mixture of antibiotics (50 μg/ml streptomycin-penicillin, 50 μg/ml gentamicin and 1.25 μg/ml amphotericin B) was used for HT29 cultivation. The pH value of supplemented MM was 7.48 ± 0.02. All media and reagents were purchased from Sigma–Aldrich. Maintenance of the cell line, between passages 145 to 149, was performed under standard conditions, at 37°C 5% CO_2_ atmosphere, in a CO2-Series Shel-Lab incubator (Sheldon Manufacturing Inc., OR, USA).

### Monitoring Behavior of HT29 in RTCA

The real time cell analyzer (RTCA-DP) xCelligence (ACEA Bioscience Inc., San Diego, CA, USA) used to monitor HT29 cells performance upon the different conditions tested, was introduced in a Heracell-240 Incubator (Thermo Electron LDD GmbH, Langenselbold, Germany) set at 37°C with 5% CO_2_ atmosphere. This technology records variations in impedance due to the adhesion, growth and morphological changes of HT29 cells during interaction with gold-microelectrodes placed in the bottom of specific microtiter plates (E-plates). The impedance signal is converted in the arbitrary “cell index” (CI) unit which is recorded in the external computer allowing, as well, data analyses through the RTCA software 1.2.1 (ACEA Bioscience).

The method to monitor the damage caused by *C. difficile* toxins was previously described by Valdés et al. (2015). In short, 16-well E-plates were seeded with 2 × 10^5^ HT29 cells (in 100 μl) and monitored (recording signal every 15 min) for 22 h to ensure the formation of a monolayer (confluent state). Afterward, the medium was removed and 200 μl of the different bacterial NCFS were added per well. Additionally, wells containing 200 μl of a control without bacteria but with Tox-S (added at 2.5% in MM, cytotoxic control) or 200 μl MM medium without bacteria or Tox-S added (non-cytotoxic control) were included in each experiment. The monitoring continued (every 10 min) for an additional 20–22 h under standard incubation conditions. CI values recorded were normalized by the time of the supernatant addition and by the control sample (MM) as previously described (Valdés et al., 2015). Samples of each bacterial supernatant were obtained from, at least, duplicated biological experiments (two independent Tox-S vs. bifidobacteria or lactobacilli incubations) and each NCFS was tested in duplicate (two independent wells within the same E-plate). Thus, four normalized-CI data were obtained per each bacterial strain tested.

### Image Analysis of HT29 Behavior

#### Time-Lapsed Monitoring in Real Time

Several images were captured in real time using the compact, inverted, optical microscope (40× objective) LumaScope-600 Series (Etaluma, Carlsbad, CA, USA) which was placed inside the Heracell-240 incubator. Images were recordered in an external computer with the software LumaView600Cy 13.7.17.0 (Etaluma). To this end, 2-well μ-Slide (ibiTreat, 1.5 polymer coverslip, tissue culture treated, sterilized slides, Ibidi GmbH, Martinsried, Germany) were seeded with 2 × 10^6^ HT29 cells/ml (1 ml) and placed on top of the microscope objective. Images were recorded every 15 min until the confluent state was reached (about 22 h); afterward, culture medium was removed and 1 ml of fresh medium containing 2.5% Tox-S or 1 ml of the NCFS collected after incubation of live *B. longum* IPLA20022 with Tox-S, was added in two independent μ-Slides. Image capture was performed for additional an 16 h.

#### End-Point CSLM Analysis

HT29 monolayers submitted to different treatments were analyzed by confocal scanning laser microscopy (CSLM) after an end-point incubation period of 20 h. For this, 8-well μ-Slide (ibiTreat, Ibidi GmbH) were seeded with 2 × 10^6^ HT29 cells/ml (0.3 ml) and incubated for 22 h to reach confluent state. Afterward, supernatant was removed and wells (in duplicate) were filled with the same volume of fresh medium containing MM (control), 2.5% Tox-S, and NCFS from live or dead *B. longum* IPLA20022 incubated with Tox-S. Incubation continued for additional 20 h; then, supernatant of each well was removed and HT29 monolayers fixed with 1 vol (0.3 ml) of cold (-20°C) acetone for 10 min. Samples were washed twice with PBS for 5 min under mild stirring and permeabilised with PBS containing 0.1% Triton 100x (Sigma) for 15 min. The nonspecific binding sites were blocked with FBS (25% in PBS) for 20 min and finally washed once with PBS. The Phalloidin-Alexa-Fluor-568 probe (Molecular Probes-Thermo Fisher, Life Technologies S.A., Madrid, Spain) toward *F*-actin was added in 0.3 ml of PBS (final concentration of 25 μl/ml) and samples were incubated overnight at 4°C in darkness. After washing twice with PBS, HT29 nucleus were stained with DAPI probe (Merck-Millipore Cor., Billerica, MA, USA) used at 1:1000 (final dilution in PBS) and incubated under the same conditions for, at least, 6 h. Finally, samples were washed and added to 0.3 ml of PBS previous visualization under microscope.

For the CSLM analysis the Leica TCS AOBS SP8 X confocal microscopy (Leica Microsystems GmbH, Heidelberg, Germany) located in the Scientific-Technical Services of Oviedo University, was used. DAPI and Alexa-Fluor-568 fluorochromes were excited at 405 nm by a blue–violet laser diode and at 578 nm by a white light laser, respectively. *Z*-stacks of HT29 samples were acquired using a 63x/1.4 oil objective applying a line average of 2 to reduce noise on the final images and a z-step of 1 micron. Details of a region were later acquired using a 2.50 optical zoom. Image-captures were recorded with the “Leica Application Suite X” software version 1.8.1.13759 (Leica).

### Statistical Analysis

To assess differences in the response (normalized CI) of HT29 due to the anti-toxin activity of *B. longum* IPLA20022, one-way ANOVA followed by SNK (Student-Newman–Keuls, *p* < 0.05) mean comparison tests were performed. The statistical package IBM SPSS Statistics for Window Version 22.0 (IBM Corp., Armonk, NY, USA) was used to carry out these analyses. Legend of **Figure 4** describes the comparisons made in each type of experiment.

## Results

**FIGURE 1:**
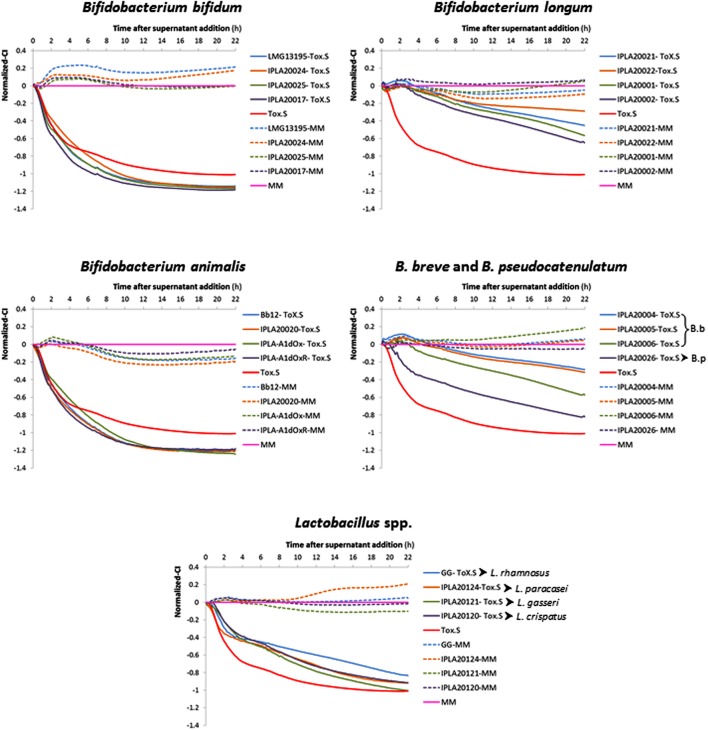
**Variation in the normalized cell index (Normalized-CI) of HT29 monolayers treated with different neutralized cell-free supernatants (NCFS; neutralized cell-free supernatants) obtained in a representative experiment after incubation of toxigenic *Clostridium difficile* supernatant (Tox-S) with different strains of *Bifidobacterium* and *Lactobacillus* species.** The Tox-S was tested alone at 2.5% (red line). Normalization was performed with respect to the point of NCFS’s addition and with respect to the control sample (culture medium MM without supernatant addition) which is the 0-reference control (pink line). The dotted lines represent results obtained with the NCFS obtained after incubation of the same strains in MM (without Tox-S). Representative SD values of these data are collected in **Table 1**.

The method previously developed by our group to detect in real time the toxic effect of *C. difficile* upon intestinal cell lines was used to address the anti-toxin probiotic potential of twenty bifidobacteria and lactobacilli strains. As an initial step several parameters were optimized in order to establish conditions for the screening using as a biological model confluent-HT29 monolayers (data no shown). Finally, neutralized (pH ≥ 7.5) cell-free supernatants (NCFS), obtained after incubation (1 h, 37°C, anaerobiosis) of each strain (about 1 × 10^9^ cfu/ml) with 5% *C. difficile* supernatant (Tox-S), were used for this study (**Supplementary Figure S1A**). The behavior of HT29 monolayers was monitored in real time recording the variations in the impedance signal (normalized-CI) over time due to the presence of the NCFS, the toxigenic control (2.5% Tox-S), or the culture media alone (MM; **Figure 1**). To understand the impedance graphs is worth noting that the lowest normalized-CI value indicates the highest toxigenic capability of *C. difficile* supernatant upon HT29; thus, in **Figure 1**, the red line (representing values obtained with 2.5% Tox-S) is the control for damage, whereas the pink line represents the non-toxigenic control (MM) used as a reference for normalization of all CI values being the reason to have “0 value.” Regarding the effect of NCFS, those obtained after incubation of strains in MM medium without *C. difficile* toxins (dotted lines) showed normalized-CI values equal or higher to the control, therefore indicating the absence of any toxic effect induced by the putative probiotics. However, when the NCFS obtained from bacteria incubated with Tox-S were analyzed, HT29 monolayers behaved differently depending on the species considered (**Figure 1**). Graphics obtained clearly show that strains belonging to species *Bifidobacterium bifidum* and *B. animalis* subsp. *lactis* had no protective effect against *C. difficile* toxins since the normalized-CI lines obtained showed similar, or even lower, values than the toxigenic Tox-S control. By contrast, the normalized-CI obtained from the four lactobacilli tested, as well as the strains of *B. longum*, *B. breve*, and *B. pseudocatenulatum* were higher than those induced by *C. difficile* supernatant. In general, normalized-CI lines from *B. longum* and *B. breve* strains were the closest to the control, thus being the strains showing higher anti-toxin capability.

Normalized-CI obtained 4 h after NCFS addition (short term effect) or 22 h after (long term effect) were analyzed in more detail (**Table 1**). Results obtained in the short term showed that all strains belonging to *B. longum* and *B. breve*, as well as *L. gasseri* IPLA20121, *L. paracasei* IPLA20124 and *L. rhamnosus* GG, seemed to have higher values of normalized-CI than the toxigenic control. However, none of the lactobacilli were able to keep the protective effect upon HT29 for a prolonged period (22 h). The strain *C. difficile* LMG21717 used in this study produced about ten-times more TcdA than TcdB (**Table 1**) and the strains showing high protective effect were those that apparently were more effectively in reducing the concentration of TcdA, i.e., belonging to *B. longum* and *B. breve* species (**Table 1**). Indeed, the NCFS obtained from strain *B. longum* IPLA20022 that promoted the lowest damage after 22 h only had 12% of remnant TcdA. Of note is that NCFS from *B. bifidum* and *B. breve* seemed to have a good ability to reduce TcdB levels (remnant between 23 and 44%), although this fact was not correlated with higher protective effect in *B. bifidum* because this species seemed to be less effective against TcdA.

**FIGURE 2:**
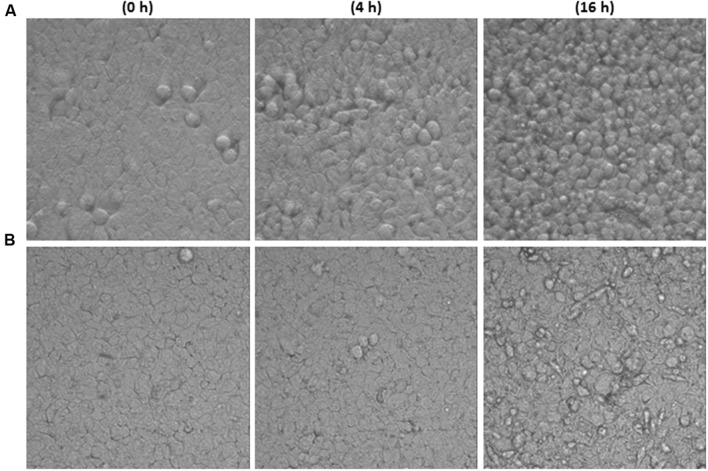
**Images of HT29 monolayers captured in real time (37°C, 5% CO_2_) with the inverted optical microscope (objective 40×) at three incubation times (0, 4, and 16 h).** Monolayer treated with toxigenic *C. difficile* supernatant (Tox-S, **A)** or with NCFS (neutralized cell-free supernatants) obtained after incubation of live *Bifidobacterium longum* IPLA20022 with Tox-S **(B)**.

**FIGURE 3:**
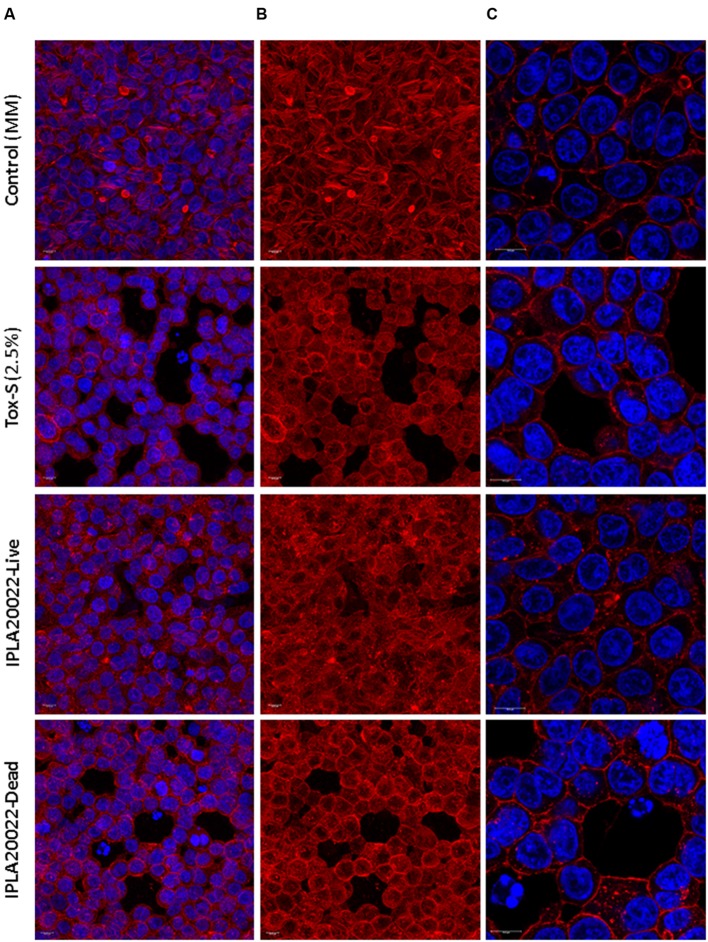
**Immunofluorescence images obtained by CSLM of HT29 after 20 h of incubation without toxigenic *C. difficile* supernatant Tox-S (control) and with Tox-S (damage control), and with NCFS (neutralized cell-free supernatants) obtained after incubation of live or dead *B. longum* IPLA20022 with Tox-S.** **(A)** and **(B)** show a *Z*-projection (thickness about 13–15 μm) of 10 XY-slides and **(C)** shows a CSLM-zoom of a XY-region. **(A)** and **(C)** show the combination of DAPI-stained nucleus (blue, excited at 405 nm by a blue–violet laser diode) and *F*-actin stained with Phalloidin-Alexa-Fluor-568 probe (red, excited at 578 nm by a white light laser); *F*-actin is also shown as a single channel in **(B)**. Bars 10 μm.

Time-lapsed microphotographs (**Figure 2**) showed that HT29 cells treated with 2.5% toxigenic *C. difficile* supernatant become spherical and the integrity of the monolayer was lost when incubation was prolonged (**Figure 2A**). However, monolayers added with NCFS from live *B. longum* IPLA20022 incubated with Tox-S remained more stable and only after a long incubation period (16 h) some cellular particles were released to the culture medium (**Figure 2B**). Furthermore, although the cytopathic mechanism of *C. difficile* toxins is well known, we performed immunohistochemistry CSLM analysis to confirm the cellular events under different treatments (**Figure 3**). Control HT29 monolayers (grown in MM for 20 h) showed a typical *F*-actin cytoskeleton in which the nucleus is imbibed, thus having an epithelial-like morphology with annexed cells well connected. However, monolayers treated for the same period with Tox-S supernatant lost the interconnection among *F*-actin filaments (**Figure 3B**) and the nucleus seems to be in the initial stages of apoptosis, i.e., the chromatin initiates the condensation showing more intense blue due to DAPI staining (**Figure 3C** and **Supplementary Figure S2**); therefore, HT29 cells become more spherical (non-epithelial morphology) and it seems that the tight junctions that maintain the monolayer integrity might have been disrupted (**Figure 3A**). The photographs obtained from HT29 monolayer treated for 20 h with the NCFS from live *B. longum* IPLA20022 were more similar to the control without toxin; the *F*-actin cytoskeleton still showed an interconnected structure and the nucleus showed less intense DAPI staining comparable to that of the negative control than the toxigenic control. This structure, resembling that of intact epithelial monolayers, is in agreement with the presence of lower amounts of remnant toxin in the NCFS and higher normalized-CI due to the capability of this strain to counteract the effect of clostridial toxins.

**FIGURE 4:**
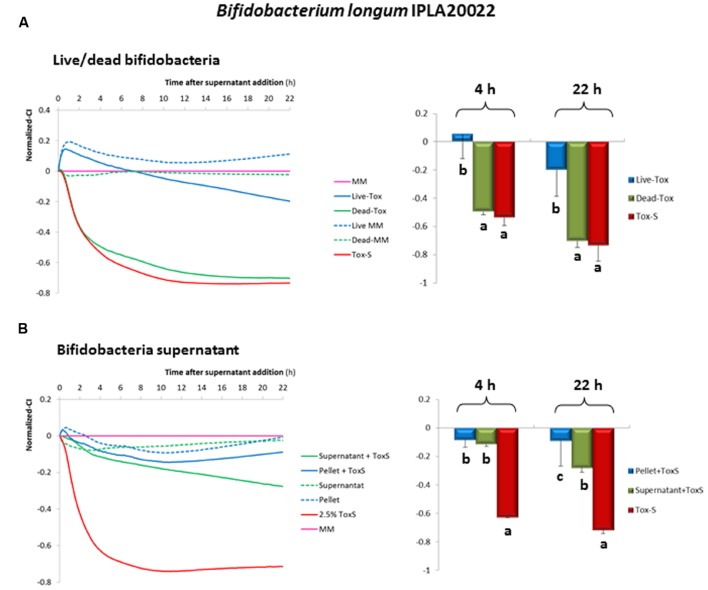
**Evolution of the normalized cell index (CI) of HT29 monolayers treated with NCFS (neutralized cell-free supernatants) obtained after incubation of toxigenic *C. difficile* supernatant (Tox-S) with live or dead *B. longum* IPLA20022 **(A)**.** Evolution of the normalized-CI of HT29 monolayers treated with 2.5% of neutralized supernatant obtained after incubation of toxigenic *C. difficile* supernatant (Tox-S) with *B. longum* IPLA20022 supernatant **(B)**. Normalization was performed with respect to the point of NCFS’s addition and with respect to the control sample (culture medium MM without supernatant addition) which is the 0-reference line (pink line). The dotted lines represent results obtained with the NCFS obtained after incubation of the live or dead *B. longum* IPLA20022 or culture supernatant in MM (without Tox-S). Histograms located in the right of **(A)** and **(B)** sections, represented the mean and standard deviation of normalized-CI values obtained at 4 and 22 h from three biological replicates each measured in duplicate; within the same time, those means that do not share a common letter are statistically different (*p* < 0.05) according to one-way ANOVA and the mean comparison SNK (Student-Newman–Keuls) test.

In order to determine whether this strain retains its anti-toxin capability under non-viable conditions, *B. longum* IPLA20022 suspension was irradiated with UV light for 90 min. The RTCA monitoring clearly showed that this treatment modified the protective effect of the bifidobacteria upon HT29 since the normalized-CI of the dead strain followed the same tendency as the toxigenic control (**Figure 4A**). Indeed, the statistical analysis performed at 4 and 22 h after NCFS addition showed that live IPLA20022 had a significantly (*p* < 0.05) higher normalized-CI, i.e., higher protective capability, than the dead strain and the toxigenic control (**Figure 4A**). Consequently, the immunohistochemistry study confirmed that the morphology of HT29 treated for 20 h with the NCFS from dead *B. longum* IPLA20022 was more similar to that obtained with the toxigenic control (**Figure 3**). Indeed, besides the *F*-actin modification, some apoptotic bodies were evidenced in both toxigenic and dead-IPLA20022 samples (**Supplementary Figure S2**) suggesting that the UV treatment of this strain, which probably affected the structure and function of the cell envelope, abolished the anti-clostridial effect of *B. longum* IPLA20022. Finally, we have tested the activity against clostridial toxins of the supernatants obtained from overnight cultures of this bifidobacterial strain. Surprisingly, the normalized-CI values were similar to those of those obtained with (live) pellets and both of them were statistically (*p* < 0.05) higher than the toxigenic control (**Figure 4B**). This result suggests that *B. longum* IPLA20022 is able to secrete factors having activity against the toxins of *C. difficile*.

## Discussion

The search for novel approaches to treat or prevent CDI is a current “hot-topic” in which the scientific community is devoting much effort. Different approaches are under investigation, most of them toward restoring the dysbiotic intestinal microbiota following infection through FMT (Youngster et al., 2014; Satokari et al., 2015) or using a consortia of defined species (Lawley et al., 2012), but also toward the application of new antibiotics (Babakhani et al., 2013; Vickers et al., 2015) and drugs to treat infections (Oresic-Bender et al., 2015), as well as vaccinations with non-toxigenic *C. difficile* strains (Senoh et al., 2015) or anti-toxin antibodies (Yang et al., 2015). Probiotic bacteriotherapy is becoming an option for the prevention of *C. difficile* recurrent infection (Leffler and Lamont, 2015), and also for the attenuation of CDI symptoms. The choice of the appropriate probiotic against *C. difficile* is of pivotal relevance since, although some formulations seem to be promising (Auclair et al., 2015), not all of them are efficient (Allen et al., 2013).

Probiotic action against CDI is based on different bacterial antagonistic mechanisms, such as competition for adhesion to gut mucosa (Banerjee et al., 2009; Zivkovic et al., 2015) and for colonization of the intestinal environment (Kondepudi et al., 2014), production of antimicrobial molecules (Schoster et al., 2013; Gebhart et al., 2015) or modulation of intestinal inflammation (Boonma et al., 2014). Another target for probiotic action is the reduction of toxicity caused by *C. difficile* (Trejo et al., 2013). In any case, if one of the active strains would be administered as a probiotic therapy to CDI patients, then the effect would only be present as long as the probiotic is consumed since stable colonization of probiotics in humans has not been shown yet.

In our study, we have explored the capability of twenty lactobacilli and bifidobacteria to counteract the effect of toxins (TcdA and TcdB) from *C. difficile* LMG21717 (equivalent to ATCC9689). The method used, based on impedance measurement of HT29 monolayers (Valdés et al., 2015), allowed a quick search of the strains showing the highest anti-toxin ability which those were belonging to *B. longum* and *B. breve* species. This fact suggests that some species-specific characteristics could account for the observed effect, although differences were also detected among strains within the same species. As far as we know, there are few comparative studies among different probiotic species; Trejo et al. (2010) co-cultivated two *C. difficile* strains (including ATCC9689) with twenty five bifidobacteria or lactobacilli and they found that the capability to antagonize the toxic effect upon Vero line (monkey fibroblast-like kidney cells) was strain dependent, but they did not report a species-efficacy association. Nevertheless, the experimental procedure used in our screening for detecting anti-toxicity was based on the incubation of the probiotic strains with a toxigenic supernatant from *C. difficile*, previous to analyze the effect of NCFS upon the biological model HT29. Then, *a priori*, the putative mechanisms that could be behind the anti-toxin capability detected with our approach are the modification of the *C. difficile* toxin and/or its availability for acting on the epithelial cells.

Some authors have reported that probiotics are able to reduce the activity of *C. difficile* toxins. Banerjee et al. (2009) observed that *Lactobacillus delbrueckii* subsp. *bulgaricus* B-30892 releases bioactive components, of unknown nature, able to decrease the toxic effect of *C. difficile* ATCC9689 upon epithelial intestinal Caco2 cells. Similarly, *Lactococcus lactis* subsp. *lactis* CIDCA8221 secretes heat-sensitive products, higher than 10 kDa, that are not affected by treatment with proteases or protease-inhibitors, which were able to protect Vero cells from *C. difficile* toxins (Bolla et al., 2013). *Saccharomyces boulardii* releases an extracellular serin-protease that was able to breakdown the toxin A, as well as to inhibit its binding to the receptor in the brush border of ileal tissue (Castagliuolo et al., 1996). In our case, analysis of the bioactivity of the supernatant collected from strain IPLA20022 directly incubated with the toxigenic *C. difficile* supernatant showed similar effect on HT29 than that obtained with the bifidobacterial pellet. Then, it seems that this strain secreted molecules able to reduce the cytotoxic effect of clostrial toxins. As far as we could find, no exo-proteases have been described for bifidobacteria and only a few peptidases have been characterized (Janer et al., 2005; Seo et al., 2007). Additionally, other molecules inducing conformational changes in proteins that disrupt the active site of other proteins, which could putatively be involved in the inactivation of *C. difficile* toxins, have been described; these are serpins (serin protein inhibitors) found in the genome of *B. longum* (Schell et al., 2002) and *B. breve* (Turroni et al., 2010) and ion chelating agents such as the iron-chelating siderophores (Cronin et al., 2012; Vazquez-Gutierrez et al., 2015). Thus, further and extensive work will be needed in order to decipher the nature of the bifidobacterial secreted factors acting against *C. difficile* toxicity.

Regarding the adsorption as mechanism to reduce toxins activity, it has been demonstrated that the soluble S-layer protein from the surface of *L. kefir* strains diminish the damage of clostridial toxins upon Vero cells, suggesting a direct interaction between the S-layer and the toxins (Carasi et al., 2012). However, as far as we could know, this type of protein cover has not been described for bifidobacteria. Additionally, cellular extracts from *L. acidophilus* GP1B were able to interfere with quorum-sensing signals from *C. difficile* and down-regulated expression of some virulence genes; both, cellular extract and *L. acidophilus* strain, were efficient in increasing the survival rate of animals in a CDI murine model (Yun et al., 2014). The lactic acid synthesized by this lactobacilli strain also had an inhibitory effect on *C. difficile* growth. Similarly, Kolling et al. (2012) reported a bactericidal effect induced by the lactic acid synthesized by *Streptococcus thermophilus* LMD-9 and, furthermore, non-inhibitory levels (10 mM) decreased the *tcdA* expression and toxin-A release. *In vivo* (CDI mouse model) treatment with live *S. thermophilus* showed a significant inverse correlation between levels of luminal lactic acid and *C. difficile* abundance in the murine gut, thus reducing the disease activity indexes of experimentation animals (Kolling et al., 2012). In our experimental design, bifidobacteria were in contact for 1 h only with the toxigenic clostridial supernatant, but not with *C. difficile*, and the putative effect of the organic acids (lactate and/or acetate) produced in this short incubation period by lactobacilli or bifidobacteria was neutralized.

Based on the results describe in this article, the adsorption of toxins to the bifidobacterial surface as well as the presence of secreted molecules responsible for the anti-toxigenic effect observed, are both plausible mechanisms of action. Nevertheless bacterial viability, which may be also needed to keep a functional bifidobacterial envelope, is required in order to maintain the anti-clostridial activity. Finally, the highest anti-toxin capability of *B. longum* and *B. breve* strains (pointing to a species-dependent efficacy) suggests that some specific characteristics of these two phylogenetically close species (Lugli et al., 2014) could account for the anti-clostridial toxicity. Further experiments must be performed in order to understand the mechanism of action behind bifidobacterial anti-*C. difficile* toxicity. Another interesting observation that will deserve further attention is the (apparently) better capability of *B. bifidum*, and to a lower extent of *B. breve*, to specifically reduce TcdB levels.

## Conclusion

In this work we have optimized a protocol to search for potential probiotics with anti-toxic activity against toxins synthesized by *C. difficile*. The impedance-based, RTCA xCelligence was a fast, reliable and efficient method for the screening of a large collection of bacteria allowing the selection of those strains with higher protection capability. In our case, strains from *B. breve* and *B. longum* showed the better performance, since they were able to reduce the levels of toxins from *C. difficile* supernatants. The best candidate to be used as probiotic to alleviate CDI was *B. longum* IPLA20022; this was the strain with the highest *in vitro* capability for reducing the levels of clostrial toxins, as well as for avoiding the cytopatic effect upon the intestinal epithelial cellular line HT29. Apart for elucidating the mechanism behind this anti-toxigenic capability, the next steps will be to study the efficacy of *B. longum* IPLA20022 in more complex *in vitro* and *in vivo* biological models before proposing its human application to treat CDI.

## Author Contributions

MG and PR-M contributed with the conception, experimental design and results interpretation of this study. LV-V carried out all experiments, OG-S advised the immunohistochemistry analysis and MA-G perform the CSLM analysis. PR-M was in charge of writing the drafted manuscript. All authors performed a critical revision of the manuscript and approved the final version.

## Conflict of Interest Statement

The authors declare that the research was conducted in the absence of any commercial or financial relationships that could be construed as a potential conflict of interest.

## Footnotes

1 http://www.ncbi.nlm.nih.gov/taxonomy/?term=Peptoclostridium%20difficile

## Supplementary Material

**FIGURE S1 | Final conditions used to perform the screening of the anti-*Clostridium difficile* cytotoxic activity upon HT29 monolayers of twenty bifidobacteria and lactobacilli strains (**A**).** Scheme of the flow-work followed to test the capability of *Bifidobacterium longum* IPLA20022 culture supernatant to act against toxigenic *C. difficile* supernatant **(B)** MRSC, MRS broth supplemented with 0.25% L-cysteine; MM, McCoy’s Medium added with supplements and antibiotics described in material and methods section; NCFS, neutralized cell-free supernatant.

**FIGURE S2 | Immunofluorescence images obtained by CSLM of HT29 after 20 h of incubation without toxigenic *C. difficile* supernatant Tox-S (control) and with Tox-S (damage control), and with NCFS (neutralized cell-free supernatants) obtained after incubation of live or dead *B. longum* IPLA20022 with Tox-S.** Images show a CSLM-2.50 optical zoom of a XY-slide. Upper-part images show the combination of DAPI-stained nucleus (blue, excite at 405 nm by a blue–violet laser diode) and *F*-actin stained with Phalloidin-Alexa-Fluor-568 probe (red, excited at 578 nm by a white light laser) **(A)**. Bottom-part images show the same magnification view at the visible (transmitted light) channel **(B)**. Arrows indicates the apoptotic bodies. Bars 10 μm.
