# Supplementary figures and images for: Screening of Bifidobacteria and Lactobacilli Able to Antagonize the Cytotoxic Effect of Clostridium difficile upon Intestinal Epithelial HT29 Monolayer
Source: Front Microbiol. 2016 Apr 22;7:577. doi: 10.3389/fmicb.2016.00577 (PMC4840286; doi:10.3389/fmicb.2016.00577)

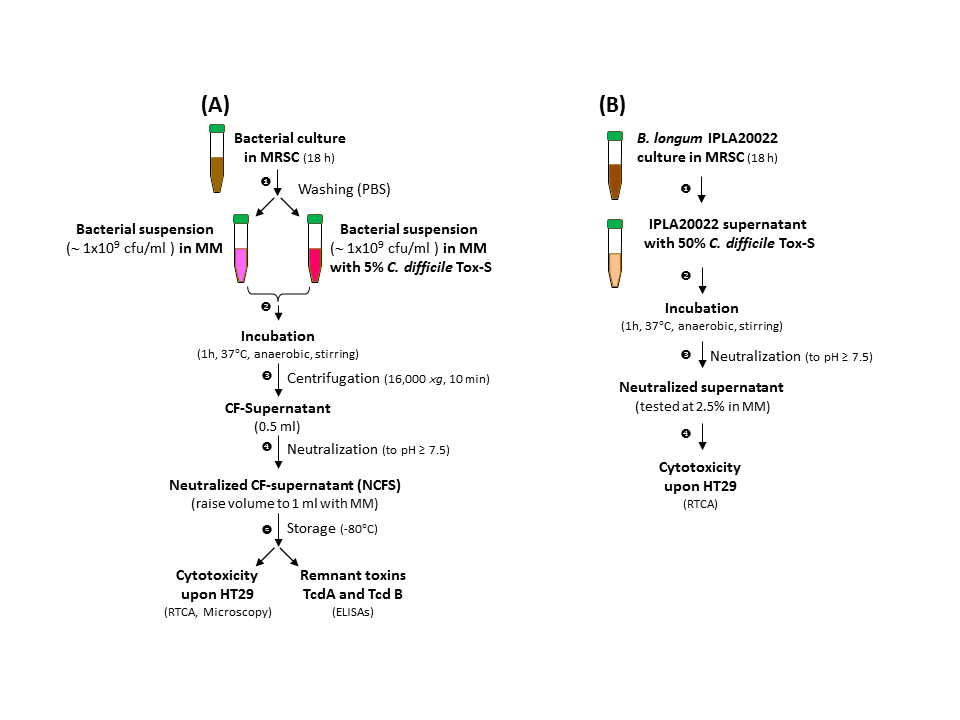

Supplement: Supplementary file 1 [file Image_1.TIF]

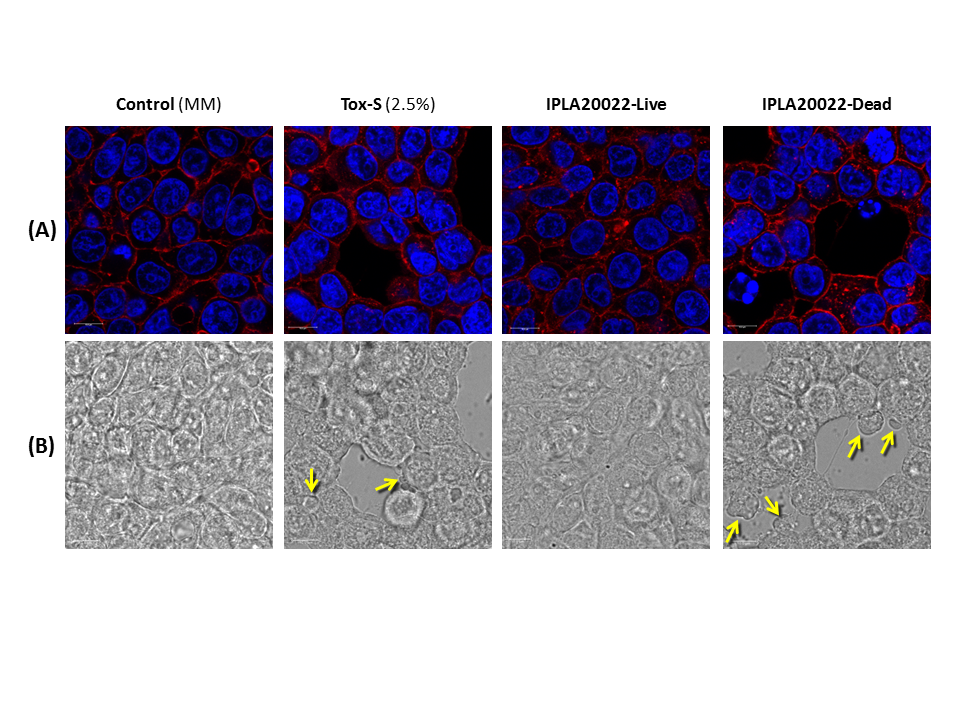

Supplement: Supplementary file 2 [file Image_2.TIF]
